# Letter from H. Crane, D. D. S.

**Published:** 1847-10

**Authors:** H. Crane

**Affiliations:** Cincinnati


Dr. Taylor:

*Dear Sir*—I understood, on my return from the East, that a resolution was passed, at the last annual meeting of the "Mississippi Association of Surgeon Dentists," to publish a "Quarterly Journal," to be devoted exclusively to the theory and practice of Dental Surgery. Now, sir, I am not informed whether any particular *modus operandi* was contemplated in that resolution. If the Association has fully matured a plan, without having given to its principal agents discretionary power, I need not have troubled you with this communication. But if such power do exist, I beg leave to make some suggestions that have presented themselves to my mind.

That a publication devoted exclusively to the theory and practice of dentistry, will be of great service to the profession, and indirectly to the public, no one can deny who is acquainted with the difficulty of obtaining practical information upon this subject. But the public has an interest in this matter that should not be overlooked, and, it will not be denied, that the profession generally are justly censurable for a dereliction of duty in not having, long since, spread documents before the public containing such information as would result in saving teeth from childhood to old age. If health, beauty, and the pleasure of perfect mastication are worth anything, they are worth more than wealth can give, and hence we discover the importance of our profession, and the responsibility resting upon those to whose care its duties are entrusted. The public then has a deep and abiding interest in this important subject, and should such information, as will avert the evils arising from a loss of the human teeth, be communicated through this source, there is not a doubt but that it will meet their hearty co-operation.

Respectfully,

Cincinnati, October 14, 1847. H. CRANE.

We hope the work will be such as to commend itself to the patronage of all who are interested in the preservation of their teeth. The Editors have thrown around them no restrictions, the object being to disseminate useful knowledge to the public as well as the profession. We regard the interest of both as identical.—Cin. Ed.

